# Anesthesia in Labor*Read at Twenty-fourth Annual Meeting of Western Texas Medical Association, San Antonio, October 25, 1900.

**Published:** 1900-11

**Authors:** S. Burg

**Affiliations:** San Antonio, Texas


### TEXAS MEDICAL JOURNAL.

ESTABLISHED JULY, 1885.

PUBLISHED MONTHLY.—SUBSCRIPTION $1.00 A YEAR.

VOL. XVI. AUSTIN, NOVEMBER, 1900. No. 5.

### Original Contributions.

For Texas Medical Journal.

### Anesthesia in Labor.*

BY S. BURG, M. D., SAN ANTONIO, TEXAS.

There is a difference of opinion among the laity, as well as the profession, as to the advisability and the extent of use of anesthesia in labor. The question arises, whether anesthesia should be used in labor not only for surgical purposes, but also as an obstetrical anesthesia—that is, as a means to lessen the pains and the distress of labor. In order to come to a definite conclusion, we have to see (1) whether all the physiological phenomena of anesthesia are the same in the conditions of labor, and (2) whether the results of a practical experience justify its employment. We all know that anesthesia is not void of danger. As in the condition of pregnancy at full term the circulation, *i. e.*, the heart action as well as the respiration have to overcome impediments, which require more energy than in normal conditions, and as the kidneys are in a state of hyperactivity, one would naturally conclude that anesthesia is more dangerous in parturition than otherwise. This theoretical reasoning is the cause why anesthesia is not so generally employed in labor. Here, as so often, we find theory and practice not entirely in accord, at least as far as my experience goes. I consider the anesthesia one of the most important parts in the obstetrical armamentarium, and its employment for analgesic purposes not only justified, but also advisable. Experience shows that the dangers

*Read at Twenty-fourth Annual Meeting of Western Texas Medical Association, San Antonio, October 25, 1900.

of anesthesia in labor, if used with proper care and close observation of the patient and not continually to a surgical degree, are exaggerated; that contrary to the theoretical reasoning the parturient woman takes the anesthetic with more ease; that she can stand it better and for a longer time than in other conditions. In an "American Text-Book of Obstetrics" the author says: "That obstetric analgesia accomplishes a distinct gain, in so far as it spares the patient the exhausting effects of severe pain and prolonged nervous tension, cannot be doubted; nor has the obstetrician any more pleasing duty than to save the needless suffering in childbed." On the other hand, I would not like to be understood that careless and long continued use or rather abuse of anesthetics in labor is not fraught with danger. It certainly takes good judgment and tact to use so-called obstetric anesthesia in simple cases of labor to advantage. Occasions for its employment are frequently offered. Take, for instance, the case of a primipara in the first stage. She has suffered already for a good many hours the most agonizing pains, having passed a sleepless night, and feels exhausted. On examination, we find the cervix very little dilated. The pains are short and frequent, recurring every two or five minutes, with no advance. The heart action is excited, the pulse being frequent and small. If you administer an anesthetic and give her an hour's rest or more, pushing it occasionally to the surgical degree, you will notice the pains becoming less frequent, but stronger and more effective, the pulse fuller, and the patient enjoying a good rest, which is confirmed by her after she is out of the narcosis. If you examine now, you will find the cervix much more dilated, often entirely retracted, and the head descended into the pelvic cavity. In such a case the anesthetic has not retarded, but advanced the process of parturition. Another condition, where the anesthetic is very useful and helps you avoid surgical interference is the so-called strictura cervicis or spasm of the cervix. Here the anesthetic given to the surgical degree removes the spasmodic condition and causes the cervix to retract, making dilatation or incision unnecessary.

For the purpose of a thorough examination the anesthetic is indispensable. How often are we in doubt about the cause of a protracted labor? The usual external and bimanual examination is often insufficient, especially when an occipito-posterior position is suspected. In such a case, or if any other obstacle has to be made out, the introduction of the whole hand is necessary, and this is only possible in an anesthesia to the full surgical degree. On the other hand, we often encounter cases of labor where, everything else being normal, the pains, although strong, are exceedingly excruciating and exhausting. By an anesthetic cautiously given this disagreeable feature is removed and labor carried to a finish to the satisfaction of patient and attendant.

In the second stage of labor, especially in multipara, the pains are frequently of a less excrutiating character and an anesthetic can easily be dispensed with. But more frequently they are exceedingly tormenting, and this in primipara, and the expulsive force of the uterine contractions, supported by abdominal pressure, is liable to produce extensive lacerations of the perineum. Here the anesthetic becomes useful in lessening the suffering and the risk of lacerations. In fact, every case may offer a good opportunity for the beneficial employment of anesthesia, by which the woman can be given a chance to give birth to her child without experiencing those agonies which usually make parturition a process so dreaded by her.

As to the kind of anesthetic to be used the opinions of the profession differ widely. Although a good many authors claim advantage for ether, as being less dangerous and producing prompter analgesia, chloroform seems to be more generally preferred, despite the fact that its dangers, compared to those of the former, are exceedingly greater, being five to one, or, according to others, twelve to one, presumably because it is less bulky to carry, is pleasanter to take, less exciting in the beginning, its effects more lasting after cessation of the inhalation. Chloral hydrate is advocated by a good many as very useful and less dangerous, but is admittedly not so analgesic as chloroform. Should then any surgical interference be required, chloroform or ether will have to be resorted to, and such a combination of the effects of the chloral and the other anesthetics is said to be dangerous. I, person lly, have no experience with chloral in labor.

After effects from anesthesia are not to be feared. Hemorrhage in the third stage of labor, because of relaxation of the uterus, is not more frequent than usual in tedious and protracted cases. Cautious and proper managmeent of this stage will prevent any disagreeable occurrence from that cause.

The possibility of the child being affected by the anesthetic, or being also anesthetised cannot be excluded, but this I have never seen to occur, except in eclampsia, or in cases with operative interference, where surgical anesthesia of several hours duration had preceded.

Medullar anesthesia, that is, the injection of a cocaine solution into the spinal canal, between the fourth and fifth lumbar vertebra (ten to fifteen minims of a two per cent. solution), has lately been reported and highly praised. By this procedure anesthesia and analgesia is produced in the lower part of the body, lower extremities and genital tract. This method is in its experimental stage, but is not void of danger. Asepsis and antisepsis in carrying it out is absolutely required. As this is not always present, at least in regard to the patient and surroundings, the possibility of injection with consequent spinal meningitis and sepsis cannot be excluded. It is not impossible though that with a more developed technique this method may replace general anesthesia in labor, as well as in other operations on the lower part of the body.

